# Novel co-culture model of T cells and midbrain organoids for investigating neurodegeneration in Parkinson’s disease

**DOI:** 10.1038/s41531-025-00882-8

**Published:** 2025-02-28

**Authors:** Elizaveta Gerasimova, Amke C. Beenen, Daniil Kachkin, Martin Regensburger, Sebastian Zundler, David B. Blumenthal, Gloria Lutzny-Geier, Beate Winner, Iryna Prots

**Affiliations:** 1 Dental Clinic 1—Department of Operative Dentistry and Periodontology, University Hospital Erlangen, Friedrich-Alexander-Universität Erlangen-Nürnberg, Erlangen, Germany; 2 Department of Stem Cell Biology, University Hospital Erlangen, Friedrich-Alexander-Universität Erlangen-Nürnberg, Erlangen, Germany; 3 Department of Molecular Neurology, University Hospital Erlangen, Friedrich-Alexander-Universität Erlangen-Nürnberg, Erlangen, Germany; 4 Center for Rare Diseases Erlangen (ZSEER), University Hospital Erlangen, FAU Erlangen-Nürnberg, Erlangen, Germany; 5 Department of Medicine 1, Translational Research Center (TRC), University Hospital Erlangen, Friedrich-Alexander-Universität Erlangen-Nürnberg, Erlangen, Germany; 6 Biomedical Network Science Lab, Department of Artificial Intelligence in Biomedical Engineering (AIBE), Friedrich-Alexander-Universität Erlangen-Nürnberg, Erlangen, Germany; 7 Department of Internal Medicine 5 - Hematology and Oncology, University Hospital Erlangen, Friedrich-Alexander-Universität Erlangen-Nürnberg, Erlangen, Germany; 8 Bavarian Cancer Research Center (BZKF), Erlangen, Germany

**Keywords:** Parkinson's disease, Parkinson's disease, Induced pluripotent stem cells, Neuroimmunology, Parkinson's disease

## Abstract

Recent studies demonstrate that brain infiltration of peripheral immune cells and their interaction with brain-resident cells contribute to Parkinson’s disease (PD). However, mechanisms of T cell-brain cell communication are not fully elucidated and models allowing investigation of interaction between T cells and brain-resident cells are required. In this study, we developed a three-dimensional (3D) model composed of stem cell-derived human midbrain organoids (hMO) and peripheral blood T cells. We demonstrated that organoids consist of multiple midbrain-specific cell types, allowing to study T cell motility and interactions with midbrain tissue in a spatially organized microenvironment. We optimized co-culture conditions and demonstrated that T cells infiltrate hMO tissue, leading to neural cell loss. Our work establishes a novel 3D cell co-culture model as a promising tool to investigate the effect of the adaptive immune system on the midbrain and can be used in future studies to address these processes in the context of PD.

## Introduction

Although for a long time, the central nervous system (CNS) was thought to be an immune-privileged organ, nowadays a number of paradigm-breaking studies demonstrated a presence of immune cells in the CNS in both, healthy state and disease conditions, including stroke, epilepsy, and neurodegenerative disorders^1–9^.

The impaired integrity of the blood-brain barrier (BBB) during neurodegeneration opens a path for immune cell infiltration^10–14^. Indeed, T cells are evident to be present in the CNS in neurodegenerative diseases, including Parkinson’s disease (PD), Alzheimer’s disease (AD), and multiple sclerosis (MS) and are suggested to contribute to disease onset and/or progression^4,15–18^. The role of T cells in neurodegeneration is further supported by studies in animal models^4,7,19–21^ and human tissue^4,6,22,23^. In neurodegeneration, T cells are suggested to exert neuronal cell damage. In line, human in vitro models revealed a T cell-neuron crosstalk and T cell-induced neuronal death^8,9,24,25^.

Growing evidence suggests that T cell infiltration into the CNS, especially injured or diseased, is not a result of passive extravasation, but a well-directed process. In order to study the mechanisms of T cell recruitment into the CNS and T cell effect on neuronal tissue in a spatiotemporal manner, suitable human models are needed. Recent advances in human induced pluripotent stem cell (hiPSC) technology provide a promising approach to bridge the gap between animal models and human diseases. The development of a new class of hiPSC-derived three-dimensional (3D) in vitro models, called organoids, counts today as a major technological breakthrough in stem cell research. The complex 3D structure of organoids resembles tissue- and organ-organization and consists of multiple tissue-/organ- or their region-specific cell types, which allow studying functional interactions in a spatially organized microenvironment that cannot be recapitulated by traditional 2D in vitro experiments.

While T cells are known to play a role in the pathophysiology of several neurodegenerative diseases, mechanisms underlying T cell interactions within the brain in health and disease, including PD remain underexplored. To address this gap, we aimed to establish human co-culture model to explore the spatially organized interactions of T cells with PD-relevant brain regions. Our approach utilizes human midbrain organoids (hMO) and peripheral blood T cells to create an in vitro co-culture system that allows investigation of T cell-neuron interactions in a physiologically relevant environment. The choice of hMO is particularly pertinent due to the midbrain’s vulnerability to neurodegeneration in PD, providing a robust platform for future studies of PD-related mechanisms and potential treatments.

To establish a functional co-culture model, we tested and optimized different conditions for co-culturing T cells with hMO to support the viability of these cell types. We demonstrated that T cells migrate into one-month old hMO and preferentially localize in the proximity of MAP2+ neurons. Our findings reveal that T cell infiltration led to notable biological changes within the hMO, including increased cell death and reduced MAP2 signal, suggesting that T cells can influence neurons in hMO and supporting the model’s utility for studying T cell effects on midbrain tissue. In contrast, we observed minimal T cell migration and T cell-mediated effects in human cortical organoids (hCO), suggesting that the midbrain is particularly susceptible to T cells, potentially relevant to midbrain vulnerability in PD. Moreover, T cell migration was enhanced in two-month-old hMO, suggesting that more aged organoids are especially relevant for investigating the link between the immune system and aging-related brain disorders. These findings collectively underscore the model’s potential for advancing the study of region-specific immune-neural interactions in the midbrain.

The 3D T cells-brain organoid co-culture model will take neurodegenerative research to a new level, allowing to study T cell recruitment and infiltration mechanisms as well as T cell spatiotemporal interactions within complex neural tissue. The 3D co-culture system, developed in this study, is a promising tool to make progress towards investigating the effects of the adaptive immune system on neural tissue and composition on a organ-like spatial level in health and disease.

## Results

### Differentiation and characterization of hMO

**Fig. 1:**
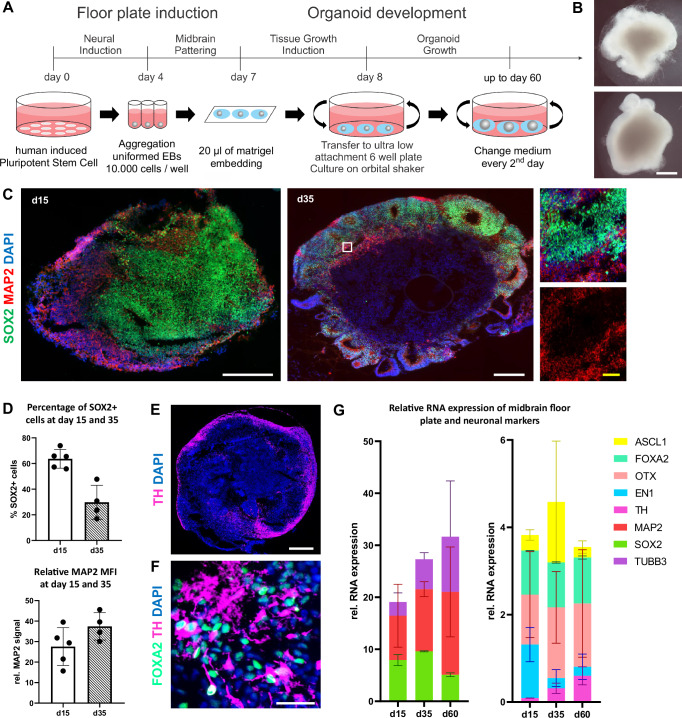
hMO express neuronal markers and have midbrain identity. **A** Schematic diagram illustrating the overall strategy of hMO generation. Diagram created by the authors using Microsoft PowerPoint. **B** Representative bright field images of hMO generated from hiPSC lines from two control individuals for 30 days. At day 30, hMO grew to more than 1 mm in diameter and extruded neuroectodermal buds. Scale bar = 500 μm. **C** hMO express neuronal and neuroprogenitor markers. Left and middle: representative immunostaining of SOX2 (green), MAP2 (red), and DAPI (blue) in hMO cryosections at days 15 (d15) and 35 (d35). Right: A zoom-in view of the white box. White scale bars = 200 μm. Yellow scale bar = 20 μm. **D** Quantification of SOX2+ cells (%) and relative MAP2 signals (Mean Fluorescence Intensity [MFI]) at days 15 (d15) and 35 (d35) in hMO cryosections. **E** TH+ cells are localized in the outer layers of hMO. Representative immunostaining of TH (purple) and DAPI (blue) in hMO cryosection at day 60. Scale bar = 500 μm. **F** hMO have midbrain identity. Representative immunostaining of FOXA2 (turquoise), TH (purple), and DAPI (blue) of apical region of hMO cryosection at day 35. TH+ cells have neuronal morphology. Scale bar = 20 μm. **G** hMO express neuronal markers, midbrain floor plate markers, and midbrain dopaminergic markers. Left: Relative transcript expression of SOX2 (green), MAP2 (red), and β3-tubulin (TUBB3, violet) in two control hMO at day 15 (d15), day 35 (d35), and day 60 (d60). Right: Relative RNA expression of ASCL1 (yellow), FOXA2 (turquoise), OTX (pink), EN1 (light blue) and TH (purple) in hMO at day 15 (d15), day 35 (d35) and day 60 (d60). Transcript expression of every marker is normalized to housekeeping genes (HKGs: RPL0, GAPDH, B2M). Bar graphs are stacked to improve clarity.

HMO were generated from healthy donors via reprogramming of fibroblasts to hiPSCs. HiPSC lines with a more than 95% positivity for a TRA1-60 pluripotency marker were differentiated into hMO for 30 and 60 days by using a guided self-organizing principle (Fig. 1A).

**Fig. 2:**
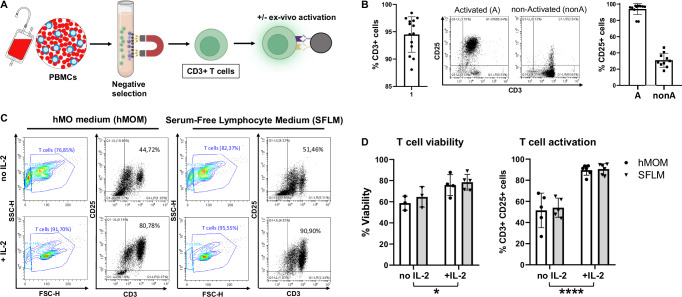
IL-2 supports T cell viability and sustained activation in co-culture media. **A** Schematic diagram illustrating the strategy of T cell isolation from human peripheral blood mononuclear cells (PBMCs) and polyclonal activation using anti-CD3/CD28-coupled dynabeads. Some parts of the diagram created with BioRender.com under a licensed academic agreement. Winner, B. (2024), and some created in Microsoft PowerPoint. **B** Validation of purity and activation of T cells prior to co-culture with organoids. Left: Successful T cell isolation was revealed by roughly 95% of CD3+ cells. Middle: Efficiency of ex vivo T cell activation determined by CD25 expression in non-activated and activated T cells after 48 hours (h) of incubation with CD3/CD28 beads. “Activated (A)”: T cells were activated for 48 h using CD3/CD28 dynabeads. “non-activated (nonA)”: T cells were incubated for 48 h without activation stimuli in the presence of IL-2. Right: Successful activation of T cells was confirmed by around 100% of activated (A) T cells expressing CD25 in contrast to non-Activated (nonA) T cells. **C** Flow cytometry-based T cell viability and activation assessment in different conditions. Contour and Dot plots of T cells cultured for 7 days with CD3/CD28 beads in hMO medium (hMOM) or Serum-Free Lymphocyte Medium (SFLM) with or without IL-2. **D** Quantification of T cell viability (live/dead staining) and activation (CD3+/CD25+ double positivity) demonstrates that in both media, IL-2 supports a more sustained activation and survival of T cells. *p < 0.05, ****p < 0.00005, two-way ANOVA.

At day 30, hMO grew to more than 1 mm in diameter spheres and extruded neuroectodermal buds (Fig. 1B), reaching a size of about 2-3 mm at day 60 (data not shown). To evaluate the neuronal and midbrain identity of generated hMO during their maturation, organoids produced from two control hiPSC lines were harvested at days 15, 35, and 60 and characterized for respective marker expression on transcriptional and protein levels. At day 35, organoids developed features of organization similar to the midbrain floor plate, namely ventricular like zones, containing SOX2+ neuroprogenitors, and mantle layers, containing maturing MAP2+ neurons (Fig. 1C). The percentage of SOX2+ cells decreased from 63.66% (±3.2% SEM) at day 15 to 29.77% (±6.6% SEM) at day 35 (Fig. 2C, D). Conversely, the proportion of relative MAP2 signal increased from 27.6% (±4.1% SEM) to 37.4% (±3.3% SEM) at day 35 (Fig. 1C, D). These data indicate that cells in the hMO gradually transitioned from proliferating SOX2+ neuroprogenitors to mature MAP2+ neurons. TH+ dopaminergic neurons were detected in the outer layers of hMO starting at day 35 alongside with FOXA2+ dopaminergic neuroprogenitors and were more abundantly present in organoids at day 60 (Fig. 1E, F).

The quantitative real-time (qRT)-PCR analysis confirmed a decrease in the expression of the neuroprogenitor marker SOX2 from day 15 to day 60, alongside an increase in the expression of neuronal markers MAP2 and β3-tubulin (Fig. 1G). Furthermore, hMO were shown to express midbrain floor plate markers including TH, FOXA2, OTX, EN1, and ASCL1. During the same period (day 15 to day 60), there was an increase in the expression of the dopaminergic neuron marker TH, while the expression of the dopaminergic progenitor marker EN1 decreased (Fig. 1G). Conversely, the expression levels of the mesodiencephalic dopaminergic neuron marker OTX and dopaminergic neuroprogenitor marker FOXA2 remained relatively stable (Fig. 1G). These findings suggest a progression of cells within hMO from dopaminergic neuroprogenitors to mature dopaminergic neurons by day 35 and day 60, while retaining some cells in the progenitor stage. Overall, neuronal differentiation was comparably effective in all organoids and expression of specific markers confirmed the midbrain cell fate including signatures of dopaminergic neurons of the substantia nigra.

### Interleukin 2 (IL-2) is essential to support T cell viability and optimal activation in co-culture conditions

T cells were isolated from peripheral blood mononuclear cells (PBMCs) of healthy donors by using a negative selection method based on a magnetic separation followed by ex vivo polyclonal activation using anti-CD3/CD28-coupled beads (Fig. 2A). Validation of isolation purity and ex vivo activation of T cells was performed by flow cytometry analysis showing roughly 95% of CD3+ cells and around 100% of CD25+ activated T cells (Fig. 2B).

Next, we aimed to determine optimal co-culture conditions, which would support a survival of hMO and a sustained viability and activation of T cells. This is technically challenging due to strong differences in classical cell culture conditions for hMO and T cells. Thus, we first cultured hMO and T cells separately for the planned co-culture duration in four different conditions, using either known optimal media or supplementing the media with known survival factors for hMO or T cells: hMO medium (hMOM), hMOM with interleukin-2 (IL-2), Serum-Free Lymphocyte Medium (SFLM), and SFLM with IL-2. hMOM provides ideal condition for hMO viability. Since hMO are usually cultured in a serum-free conditions, SFLM (developed specifically for serum-free T cell expansion) might be compatible with organoid culture. IL-2 is well-documented for its role in T cell growth and survival; thus, we tested IL-2 in both, hMOM and SFLM to support T cell viability and activation in the co-culture.

Following T cell culture without hMO for 7 days in four conditions, described above, T cells were profiled by flow cytometry for their viability and activation status. As expected, IL-2 was essential for maintaining T cell viability and activation regardless of the medium being used. Flow cytometry analysis demonstrated enhanced cell granularity and more debris in cultures without IL-2 in both, hMOM and SFLM, reflecting decreased T cell viability (Fig. 2C, forward/side scatter [FSC/SSC], and 2D). Moreover, as expected, IL-2 supported a more sustained activation of T cells during culture in both media as determined by the frequency of CD25+/CD3+ double positive cells (Fig. 2C, D). Thus, the optimal co-culture condition for T cells contains media supplemented with IL-2.

### hMOM is the optimal medium for hMO viability

**Fig. 3:**
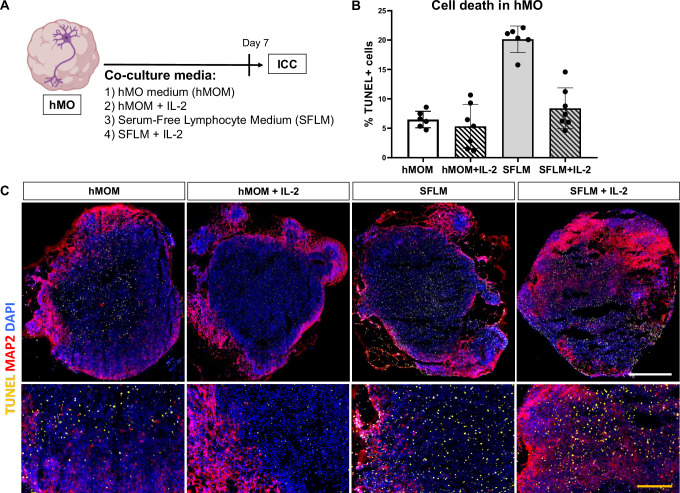
Assessment of the hMO viability in different conditions. **A** Schematic diagram illustrating the overall strategy of testing the effect of 4 different conditions on hMO. Immunocytochemistry was used for evaluation of cell death and neuronal survival by the TUNEL assay and MAP2 staining, respectively. Some parts of the diagram created with BioRender.com under a licensed academic agreement. Winner, B. (2024), and some created in Microsoft PowerPoint. **B** Quantification of cell death (% TUNEL+ cells) within organoid tissue. Increased TUNEL signal was detected in Serum-Free Lymphocyte Medium (SFLM) without IL-2, suggesting that SFLM itself causes cell death in hMO. **C** Comparison of hMO viability after culturing them for 7 days in four different conditions. Up: Representative cryosections of hMO stained for MAP2 (purple), TUNEL (yellow) and DAPI (blue). Scale bar = 500 μm. Bottom: A zoom-in view. Yellow scale bar = 100 μm.

We next analyzed cell death rate of hMO in both types of media and in the presence or absence of IL-2 (Fig. 3A). hMO were cultured in the respective media without T cells for 7 days and cell death was afterwards determined by the TUNEL immunofluorescence staining, a method for detecting apoptotic DNA fragmentation. In SFLM with and without IL-2, an increased cell death in day 30 hMO was determined (Fig. 3B, C). Notably, an extent of cell death in hMO cultures in hMOM with or without IL-2 was comparable and fluctuated around 7% (Fig. 3B), matching the usual range of basal cell death rate in human cell cultures. Importantly, irrespective of the type of medium, hMO morphology and MAP2 signal were not significantly altered (Fig. 3C).

Considering the data for both, T cells and hMO, most optimal co-culture condition to support viability of hMO and T cell activation is hMOM supplemented with IL-2. This condition was used for all consequent co-culture experiments of hMO and T cells.

### Activated T cells show enhanced infiltration and specific integrin profile in co-culture with hMO

**Fig. 4:**
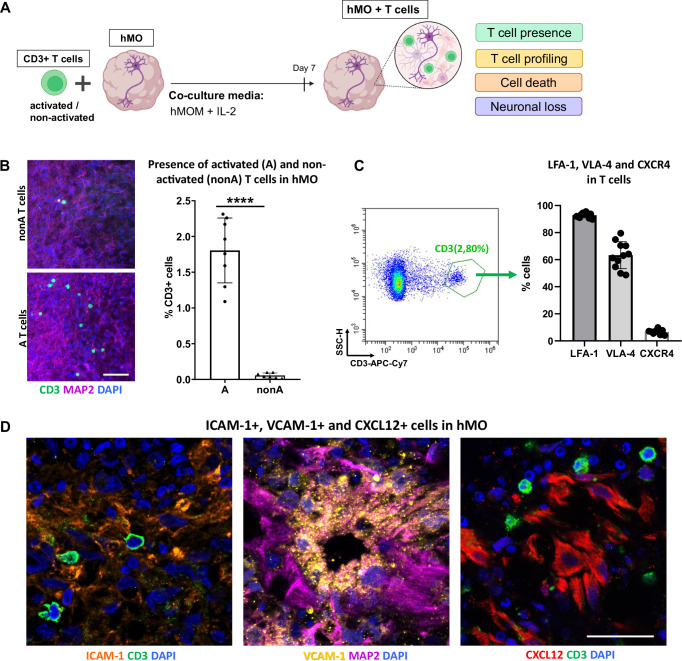
Activated T cells show enhanced infiltration and specific integrin profile in co-culture with hMO. **A** Schematic diagram illustrating the strategy of a 3D T cell-midbrain organoid co-culture system. CD3+ T cells (either ex vivo activated or non-activated) were co-cultured with hMO for 7 days in hMOM with IL-2, which determined to be optimal for the survival of both, hMO cells and T cells. After 7 days of co-culture, T cell presence, T cell subsets, cell death, and neuronal loss in hMO tissue were analyzed. Some parts of the diagram created with BioRender.com under a licensed academic agreement. Winner, B. (2024), and some created in Microsoft PowerPoint. **B** Co-culture of hMO with either activated (A) or non-activated (nonA) CD3+ T cells for 7 days resulted in T cell presence in hMO tissue with higher infiltration of activated T cells in comparison to non-activated. Left: Schematic diagram illustrating the co-culture experimental workflow. Middle: representative immunostaining of CD3 (green) and MAP2 (purple) of hMO after co-culture with nonA and A T cells. Scale bar = 50 μm. Right: Quantitative analysis of T cell infiltration, represented by % CD3+ cells, showing significantly higher infiltration of A T cells compared to nonA T cells. ****p < 0.00005, Student’s *t* test. **C** Flow cytometry-based characterization of infiltrating T cells reveals high expression of integrins LFA-1 and VLA-4 and low expression of the chemokine receptor CXCR4. Left: Representative flow cytometry dot plot demonstrating the gating strategy for infiltrating T cells and hMO cells. Right: Quantitative analysis of LFA-1-, VLA-4-, and CXCR4-expressing infiltrating CD3+ T cells. **D** Representative immunostaining of integrins and chemokine ligands in hMO tissue: ICAM-1 (orange), VCAM-1 (yellow), and CXCL12 (red). Scale bar = 20 μm.

Next, to study T cell-neuron interactions and T cell-driven effects on midbrain tissue in a spatial manner, we performed T cell-hMO co-culture (Fig. 4A). Human T cells, isolated from peripheral blood, and hMO, differentiated from hiPSC lines, were co-cultured in hMOM supplemented with IL-2, which previous experiments determined to be an optimal condition for the survival of both hMO and T cells, and for supporting T cell ex vivo activation. To determine T cell migration into hMO and the consequences of T cell presence in hMO tissue, co-cultured hMO were investigated for T cell presence, cell death, and neuronal loss (Fig. 4A).

To investigate the potential of T cells to infiltrate hMO tissue, we first co-cultured 30 days-old hMO with allogeneic T cells either ex vivo polyclonally activated or not activated. Regardless of T cell activation, all co-cultures were performed in hMOM supplemented with IL-2, where IL-2 provides necessary survival signals to T cells especially in the absence of activation. Co-culture experiments were performed at a ratio of 0.5 million T cells to one organoid, within the range of the calculated ratio in postmortem midbrain tissues of PD patients^9^. Immunocytochemistry revealed the presence of CD3+ T cells in hMO tissue after co-culture, indicating that T cells are capable of infiltrating hMO tissue in a 3D model (Fig. 4B). Moreover, significantly more activated T cells were present within the hMO compared to non-activated T cells (Fig. 4B). After co-culture, less than 0.1% of non-activated T cells had infiltrated the hMO. In contrast, approximately 1.5% of activated T cells successfully migrated into the hMO tissue, highlighting a stronger infiltration capability of activated T cells. For the subsequent co-culture experiments, only activated T cells were used.

To identify the specific molecules potentially facilitating T cell migration into hMO, we dissociated hMO tissue with infiltrating T cells and profiled infiltrated CD3+ T cells by flow cytometry for the expression of integrins and adhesion molecules (Fig. 4C and Supplementary Fig. S1). Flow cytometry analysis revealed that around 90% of infiltrating T cells were positive for LFA-1, while 60% were positive for VLA-4, and less than 10% were positive for the chemokine receptor CXCR4 (Fig. 4C, right panel). LFA-1 and VLA-4 integrins are known to support T cell adhesion and migration across tissue barriers. LFA-1- and VLA-4-positivity of hMO-infiltrating T cells indicates that they are equipped for tissue infiltration and stable interactions within the organoid microenvironment, enhancing their ability to enter and remain within neural tissue. CXCR4, a chemokine receptor commonly associated with T cell homing to lymphoid organs, has been proposed to facilitate T cell infiltration into the brains of patients with Lewy body dementia^6^. Low expression of CXCR4 on the infiltrating T cells in our model suggests that CXCR4 might contribute to T cell migration in a disease-relevant context, while our model utilizes hMO and T cells from healthy individuals. To further explore the potential involvement of adhesion molecules in the interaction between T cells and the hMO, immunostaining was performed to assess the presence of key integrin- and chemokine ligands within the hMO tissue. ICAM-1 and VCAM-1, the primary ligands for LFA-1 and VLA-4 integrins, as well as CXCL12, the chemokine ligand for CXCR4, were confirmed to be present in hMO tissue, providing adhesion sites that likely facilitate T cell retention and stable interactions within the organoid (Fig. 4D).

### Infiltration of T cells, producing cytotoxic mediators and cytokines, correlates with increased neurotoxicity in hMO

We next analyzed whether infiltration of T cells induce cell death and/or neuronal loss in hMO.

**Fig. 5:**
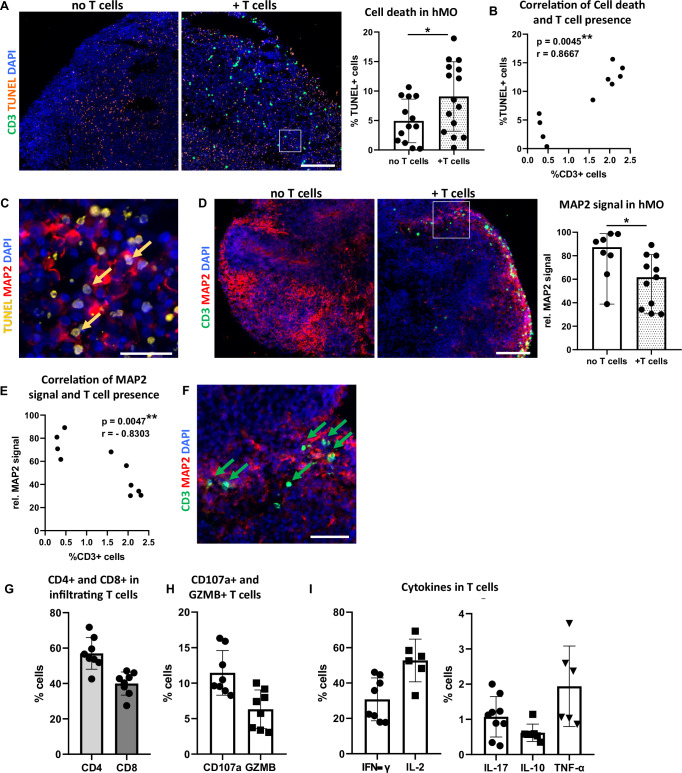
T cell infiltration correlates with cell death and neuronal loss in hMO tissue. **A** Increased cell death in hMO co-culture with T cells (+T cells) in comparison to hMO cultured without T cells (no T cells). Left: Representative immunostaining of CD3 (green) and TUNEL (orange). Scale bar = 200 μm. Right: Quantitative comparison of cell death (% TUNEL+ cells) in hMO co-cultured with or without T cells. *p < 0.05, Student’s *t* test. **B** Correlation between T cell presence and cell death in hMO tissue. Scatter plot showing the relationship between T cell presence (% CD3+ cells) and cell death (% TUNEL+ cells) in hMO. A significant correlation was observed (r = 0.8667, **p = 0.0045, Spearman’s correlation, two-tailed). **C** Majority of TUNEL signal (examples indicated by arrows) localizes in MAP2+ neuronal somas. Representative immunostaining of TUNEL (yellow) and MAP2 (red). Scale bar = 20 μm. **D** Decreased MAP2 signal in hMO co-cultured with T cells in comparison to hMO cultured without T cells. Left: Representative immunostaining of CD3 (green) and MAP2 (red). Scale bar = 200 μm. Right: Quantitative comparison of MAP2+ neurons (rel. MAP2 signal) in hMO cultured with or without T cells. *p < 0.05, Student’s *t* test. **E** Correlation between T cell presence and MAP2+ neurons in hMO tissue. Scatter plot showing the relationship between T cell presence (% CD3+ cells) and rel. MAP2 signal in hMO. A significant negative correlation was observed (r = −0.8303, **p = 0.0047, Spearman’s correlation, two-tailed). **F** T cells localize in the vicinity of MAP2+ neurons (examples indicated by arrows). Representative immunostaining of CD3 (green) and MAP2 (red). Scale bar = 50 μm. Quantitative flow cytometry analysis of **G** CD4 and CD8 fractions, **H** cytolytic and degranulation markers CD107a and granzyme B (GZMB), and **I** pro-inflammatory (IFN‐γ, IL-2, IL-17, TNF-α) and anti-inflammatory (IL-10) cytokines expressed by infiltrating T cells.

In comparison to hMO cultured without T cells, significantly more TUNEL+ cells were observed in hMO co-cultured with T cells (Fig. 5A), indicating increased cell death. A significant positive correlation was found between the percentage of T cells and the percentage of TUNEL+ cells in hMO tissue (Fig. 5B). Immunocytochemical analysis revealed that the majority of TUNEL+ cells were MAP2+ neurons, further emphasizing that neuronal cells are particularly affected (Fig. 5C). In line, co-culture with T cells led to a significant reduction in MAP2 signal in hMO, indicating neuronal loss (Fig. 5D). This observation was supported by a significant negative correlation between the percentage of migrated T cells and the relative MAP2 signal in hMO (Fig. 5E). Notably, T cells preferentially migrated toward and localized in the proximity to MAP2+ neurons (Fig. 5D, F), suggesting targeted interactions between T cells and neuronal cells within the organoid tissue.

Since T cells and iPSCs in our model derived from different individuals, we addressed a potential role of major histocompatibility complex (MHC) class I mismatches in allogeneic T cell:hMO tissue interactions in our model. To investigate this, we performed immunocytochemistry staining for MHC class I in hMO. While robust MHC class I expression was determined in (lipopolysaccharide) LPS-treated astrocytes, used as a positive control, no MHC class I signal was present in hMO tissue (Supplementary Fig. S2), suggesting that hMO do not express MHC class I. The combination of a polyclonal stimulation of T cells prior co-culture and no MHC class I expression in hMO strongly suggests that observed neuro-/cytotoxicity in our allogeneic model is mediated through MHC-independent mechanisms. To assess the cytotoxic potential of infiltrating T cells, we performed their phenotypical and functional characterization and analyzed their subtype composition as well as expression of cytotoxic mediators and cytokines. Flow cytometry analysis revealed that hMO-infiltrating T cell population included approximately 40% of CD8+ T cells and around 60% of CD4+ T cells (Fig. 5G and Supplementary Fig. S1). Notably, CD8+ T cells express cytolytic and degranulation markers, including CD107a and granzyme B (Fig. 5H), suggesting that CD8+ T cells within hMO can directly contribute to neuronal cell death through cytotoxic granule release. In addition to cytolytic markers, infiltrated T cells secreted pro-inflammatory and regulatory cytokines, including IFN-γ, IL-17, TNF-α, and IL-10 (Fig. 5I), which are known to either exacerbate neuronal damage or to modulate an immune over-reaction in inflammatory environments. The migration of both, cytotoxic (CD8+) and helper (CD4+) T cells into hMO tissue suggests that T cells may cause neuronal damage in hMO through multiple mechanisms: directly killing via cytotoxic granules and inducing pathology via cytokine-mediated effects. Together, these findings indicate cytotoxic and neurodegenerative effects of infiltrating T cells in hMO tissue and highlight that established co-culture system enables modeling T cell-driven effects on brain tissue.

### Brain region-specific and age-dependent susceptibility of hMO to T cells

**Fig. 6:**
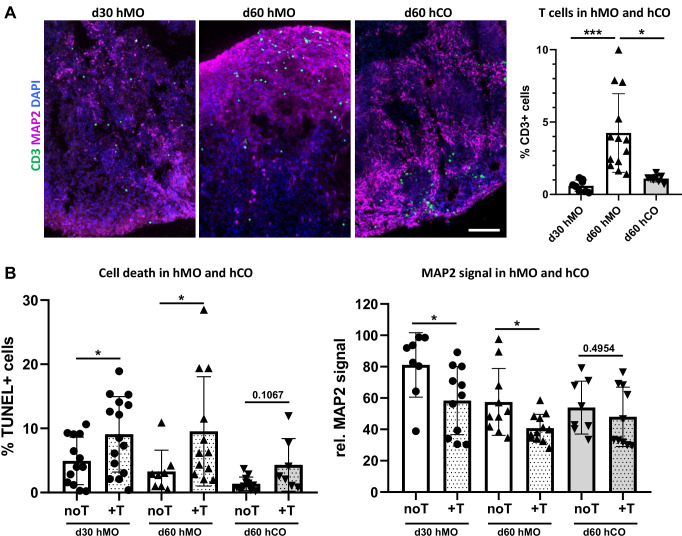
Differential T cell infiltration and neuronal cell death in hMO and hCO. **A** Increased T cell infiltration in hMO in comparison to hCO tissue. Representative immunostaining of CD3 (green) and MAP2 (purple) in 30-day-old hMO (d30 hMO), 60-day-old hMO (d60 hMO), and 60-day-old hCO (d60 hCO) after co-culture with T cells. Scale bar = 50 μm. Right: Quantitative analysis shows a significantly higher percentage of T cells infiltrating d60 hMO compared to both, d30 hMO (***p < 0.0001) and d60 hCO (*p < 0.05). **B** T cell infiltration leads to increased cell death and MAP2 decrease in hMO but not in hCO tissue. Left: Quantitative comparison indicates a significant increase in cell death (%TUNEL+ cells) in d30 and d60 hMO (*p < 0.05) after co-culture with T cells (+T), while only minimal cell death increase is observed in d60 hCO after co-culture with T cells. Right: relative MAP2 signal comparison shows decreased MAP2 expression in both, d30 and d60 hMO co-cultured with T cells, while d60 hCO MAP2 levels remain unaffected by co-culture with T cells (+T).

Next, we hypothesized that more aged hMO tissue would exhibit greater susceptibility to T cell infiltration, reflecting the potential impact of aging on T cell-neuron interactions. To test this, we co-cultured day 30 and day 60 hMO with activated T cells for 7 days. As expected, significantly more T cells were found in the day 60 (two-month-old) hMO compared to the one-month-old ones (Fig. 6A). An increased T cell presence in both one-month- and two-month-old hMO was associated with cell death and a MAP2 signal decrease (Fig. 6B). The enhanced T cell infiltration into older day 60 hMO might be especially relevant for studying PD and other age-related neurodegenerative disorders using our co-culture model.

To model T cell interaction with different brain regions, we expanded our approach by establishing a co-culture of T cells with human cerebral organoids (hCO) that in our hands demonstrate forebrain identity (Supplementary Fig. S3A–C). Using a similar method, we co-cultured T cells with day 60 hCO for 7 days in hCO medium (hCOM) supplemented with IL-2. The addition of IL-2 to the hCOM did not affect hCO and did not increase hCO cell death (Supplementary Fig. S3D), indicating that this condition is acceptable for the T cell-hCO co-culture. We demonstrated that T cells migrate into hCO tissue (Fig. 6A), indicating that the co-culture model developed in this study is adaptable and can be applied to different types of brain organoids, enabling the exploration of T cell interactions with various brain regions. Quantitative analysis revealed that significantly more T cells infiltrated day 60 hMO compared to day 60 hCO (Fig. 6A), indicating that the midbrain-like tissue is more vulnerable to T cell infiltration. In contrast to hMO, where T cell migration led to significant cell death and neuronal loss, hCO showed minimal cell death and stable MAP2 signal after co-culture with T cells, suggesting that cortical tissue may be more resistant to T cell-driven cytotoxic effects under the same co-culture conditions. Furthermore, these results demonstrate targeted and brain region-specific T cell effects, potentially depending on surrounding tissue milieu.

**Fig. 7:**
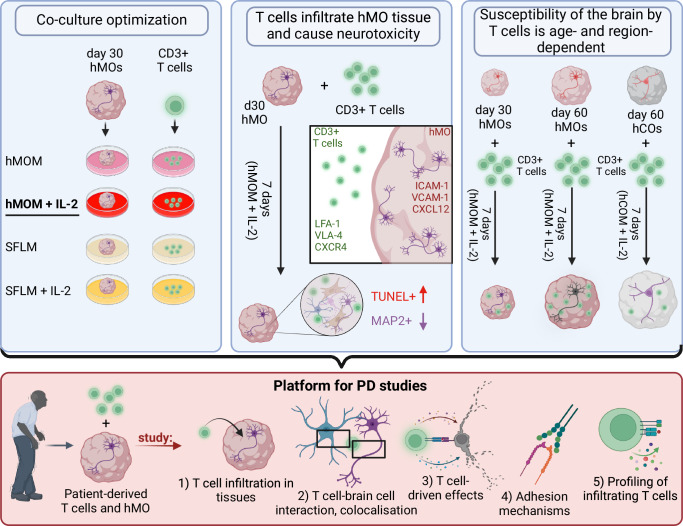
Schematic diagram illustrating the strategy of development and usage of T cell-midbrain organoid co-culture system. As a first step, different co-culture conditions were tested. hMOM with IL-2 was shown to be optimal for viability of both, hMO and T cells. As a second step, T cell infiltration into hMO and T cell-neuron interactions were studied. T cells were shown to induce cell death and MAP2+ neuronal loss. As a third step, age- and region-specific susceptibility of brain organoids to T cell infiltration was revealed. Day 60 aged hMO were shown to be more vulnerable to T cell infiltration and T cell-driven neurotoxicity. Thus, the 3D co-culture model developed in this study provides a versatile platform for investigating immune-neural interactions in PD: by incorporating PD-specific T cells and brain organoids derived from PD patients’ stem cells, it enables the examination of T cell infiltration into hMO, spatial colocalization and cell-type specificity of T cell interactions within organoid tissue, T cell-driven effects, adhesion mechanisms facilitating T cell infiltration, and functional characterization of T cells and their subsets infiltrating the organoids. Figure is created with BioRender.com under a licensed academic agreement. Winner, B. (2024) https://BioRender.com/v54o777.

In conclusion, the 3D co-culture model developed in this study provides a versatile platform for investigating T cell-neuron interactions, revealing age- and region-specific susceptibility of brain organoids to T cell infiltration and cytotoxic effects (Fig. 7).

## Discussion

In this study, we developed a 3D co-culture model of peripheral blood T cells and hMO to provide a system for investigation T cell-brain cell interactions in a spatial manner. In this study, we utilized hMO since the midbrain is particularly relevant for PD pathology and degeneration of dopaminergic neurons in the substantia nigra is a hallmark of the disease. We turn our focus here on establishing a model to study the intricate interplay between T cells and brain resident cells, as the role of T cells in PD remains understudied compared to their role in AD and MS. In particular, we generated hMO and co-cultured them with T cells to establish, optimize, and validate a co-culture model, demonstrating that T cells can indeed infiltrate and exert biological effects on human midbrain tissue. Our primary goal was to develop a functional platform that enables the evaluation of T cell-neuron or T cell-glial cell interactions and their consequences in a controlled, physiological-like, spatial 3D environment.

The uncovered ability of T cells to enter hMO tissue allows modeling aspects of their infiltration into the human brain as well as studing trafficking mechanisms and co-localization with other cell types. Shedding light on mechanisms facilitating T cell migration into hMO tissue, we demonstrated expression of important integrins (LFA-1 and VTL-4) in migrating T cells and their ligands (ICAM-1 and VCAM-1) in hMO tissue. Interestingly, despite being reported to participate in T cell brain infiltration in DLB patients^6^, only a minor percentage of infiltrating T cells in our model was expressing CXCR4. This observation aligns with the migration of these T cells into a non-lymphoid, neural tissue environment, consistent with patterns seen in activated T cells that home to inflamed or damaged tissues rather than to lymphoid structures. Moreover, the low expression of CXCR4 on infiltrating T cells in our model, composed of T cells and hMO from healthy individuals, suggests that disease-relevant mechanisms of T cell brain infiltration need to be investigated in further studies by applying of patient-derived iPSC and T cells.

In addition to T cell entry, we observed that infiltrating T cells release cytotoxic proteins (e.g., granzyme B and CD107a) and secrete pro-inflammatory cytokines such as IFN-γ, TNF-α, and IL-17, indicating that T cells may contribute to neuronal damage and/or cell death through both, cytotoxic and cytokine-mediated pathways. Indeed, after co-culture with T cells, we detected increased cell death and neuronal loss in hMO. Furthermore, a significant correlation between T cell presence and cell death, as well as its negative correlation with MAP2 signal in hMO after co-culture suggests that T cell infiltration might cause increased neuronal loss in hMO. These data align with previous reports of T cell-mediated neurodegeneration and will enable deeper investigation of T cell-mediated neurodegenerative processes occurring in PD and other neurodegenerative disorders, which may be triggered and/or exacerbated by adaptive immune cells^4,6,9,26^. Thus, presented in this study co-culture model allows to study T cell-mediated effects on brain tissue and the mechanisms of T cell-driven neurotoxicity in a more physiologically-relevant, 3D spatial brain tissue context that (from available cell culture models) most closely mimics human in vivo environment.

While MHC mismatch could theoretically contribute to T cell activation and graft-versus-host response in the allogeneic co-culture of T cells and hMO from different individuals, all T cells in our model were polyclonally activated with CD3/CD28 beads prior to co-culture, bypassing MHC-antigen-T cell receptor (TCR) binding. Their activation, therefore, is not based on MHC-TCR interaction and T cell-mediated cytotoxicity is primarily attributed to cytolytic mediators and cytokines. Additionally, the absence of professional antigen-presenting cells and faint (if any) MHC class I expression in iPSC-derived neurons and hMO, shown by us and others^9,27,28^, reduces the likelihood of cytotoxicity being mediated by classical graft-versus-host responses. In line, previous studies have shown that polyclonally activated T cells can mediate cytotoxicity in both, allogeneic and syngeneic systems through MHC-independent mechanisms^28,29^.

Our co-culture model demonstrated significant versatility and adaptability. Initially, we developed and optimized the conditions to co-culture T cells with midbrain organoids using one-month-old hMO. We then successfully extended this model to establish co-cultures with two-month-old hMO. We observed significantly higher infiltration of T cells into more mature, two-month-old hMO compared to one-month-old hMO, indicating that aged midbrain tissues may be more susceptible to T cell infiltration and subsequent T cell-driven damage. An increased vulnerability of aged hMO is especially relevant to future studies of age-related neurodegenerative disorders such as PD.

Additionally, we extended our approach to co-culture T cells with hCO as a model for different brain regions. Our findings showed that T cells infiltrate hCO tissue but to a significantly lesser extent than hMO. Moreover, unlike in hMO, T cell infiltration in hCO did not lead to increased cell death or MAP2 reduction, suggesting that hMO tissue is more vulnerable to T cell-driven cytotoxic effects. This finding is in line with previous studies showing that dopaminergic neurons are particularly vulnerable due to their high levels of reactive dopamine, metabolic demands, and mitochondrial turnover^30–33^. Furthermore, the adaptability of our model highlights its potential for studying brain region-specific immune-neural interactions.

We and others demonstrated previously that T cells are evident the midbrain in PD patients^4,9^, and established in this study model is a powerful tool to study the direct effects of T cells on midbrain neural cells. Despite the significant advantages of our 3D co-culture model, it does not contain the BBB mechanisms, which control immune cell entry into the CNS. To address this, future developments could utilize vascularized brain organoids, incorporating endothelial cells and displaying more distinct BBB characteristics^34,35^ or BBB assembloids, constructed from brain and blood vessel organoids^36^. These developments would allow to study T cell migration across the BBB in the context of the disease. Additionally, incorporating microglia, the resident immune cells of the CNS, into the brain organoids^37–40^ would further enhance relevance of the co-culture model for studying neuroinflammatory responses and microglia-dependent T cell effects within the brain.

While our results show that T cells can migrate into hMO tissue and induce neuronal loss, it is important to mention that this study utilizes cells from healthy donors, not PD-specific cells. Therefore, our results do not suggest any PD-specific mechanisms and do not confirm that T cells drive PD pathology. Instead, our model lays the groundwork for future studies that can incorporate disease-specific T cells and brain organoids from PD patients’ stem cells to more accurately model PD pathology and investigate underlying immune-related processes. Our model offers a unique advantage by allowing investigation of spatial interactions between pathogenically-relevant T cells, neurons, and glial cells in midbrain organoids harboring disease-causing mutations or derived from patients’ iPSCs. These organoids are shown to exert PD-relevant phenotypes, including fewer and less complex midbrain dopaminergic neurons, as well as increased aggregation and abnormal clearance of α-synuclein^41–44^. Moreover, gene expression profile of PD midbrain organoids has significant similarities with that of PD patients’ brain tissue^44,45^. Therefore, our co-culture model using hMO is indeed a versatile tool for investigating the role of T cells in neurodegenerative diseases and enables investigation of different aspects of T cell involvement in PD pathology.

In conclusion, our study provides a novel 3D co-culture technology that allows detailed investigation of T cell-neural tissue interactions. The increased vulnerability of hMO to T cell entry and the associated neurotoxic effects highlights the need for further study of regional brain differences in T cell susceptibility. Our model provides a foundation for future advancements, such as utilizing disease-specific T cells and organoids and introducing the BBB and microglial cells to the system. Our adaptable co-culture model offers a promising avenue for shedding light on adaptive immune system contribution to neurodegenerative diseases and for developing targeted therapeutic strategies to modulate immune responses in the CNS.

## Methods

### Generation and cultivation of hMO and hCO

HiPSC were reprogrammed using retroviral transduction of the transcription factors OCT3/4, c-MYC, SOX2, and KLF4 as previously described (Havlicek et al., 2014). All the hiPSCs lines were screened for pluripotency and for stable karyotype using the G-banding chromosomal analysis. The study was performed in accordance with the Declaration of Helsinki. All experiments using hiPSC-derived cells and peripheral blood T cells were carried out in accordance with the local Institutional Review Board approval (No. 4120 and No. 259_17B, University Hospital Erlangen, FAU Erlangen-Nürnberg, Erlangen, Germany), as well as national and European Union directives. Written informed consent was received from voluntary donors of skin biopsies and blood donations prior to inclusion in the study at the Movement Disorder Clinic at the Department of Molecular Neurology, University Hospital Erlangen.

HiPSC were cultured on plates coated with Geltrex (Thermo Fisher) in mTeSR Plus media (StemCell Technologies) with 1% Penicillin/Streptomycin (P/S) at 37°C with 5% CO_2_ with daily media change. To maintain the hiPSC culture, the cells were split upon reaching a confluence of ~70% using ReLeSR (StemCell Technologies). ReLeSR was applied for 7 minutes (min) at room temperature (RT). Following incubation with ReLeSR, the undifferentiated hiPSC colony starts to lift from the culture plate, while differentiated cells remain attached. After shaking/tapping of the culture plate, that allows undifferentiated cells completely lift off and broke up into optimally sized aggregates for replating, fresh mTeSR was applied. HiPSC cell aggregates were resuspended and split at a 1:10 ratio.

HiPSC lines of two healthy control female Caucasian individuals with no evidence of neurological disease (UKER-i33Q-S1-006 and UKER-i7MN-S1-010) were differentiated into hMO as previously described^46^. In brief, hiPSC were dissociated with Accutase (Life Technologies) to single cells and were plated (10^4^ cells/well) in low-attachment, U-shaped 96-well plates (Costar) with 200 µl/well of neuronal induction medium (DMEM/F12:Neurobasal [1:1, Life Technologies], supplemented with N2 supplement [Invitrogen], B27 without vitamin A [Invitrogen], 1% GlutaMAX [Invitrogen], 1% minimum essential media-nonessential amino acid [Invitrogen], 0.1% β-mercaptoethanol [Invitrogen], 1 μg/ml Heparin [Sigma-Aldrich], 10 μM SB431542 [Stemgent], 200 ng/ml Noggin [Prospec], 0.8 μM CHIR99021 [Reagents Direct]) supplemented with 10 μM ROCK inhibitor Y27632 (Calbiochem). The ROCK inhibitor was added for the first 48 h, and the neuronal induction medium was changed on day 2. On day 4, hMO were cultured with the addition of 100 ng/ml SHH-C25II (R&D Systems) and 100 ng/ml FGF8 (R&D Systems) for midbrain patterning. Three days later, when neuroectodermal buds were starting to extrude, the hMO were embedded in 20 μL of Matrigel (Corning) and cultured for 24 h in tissue growth induction medium containing Neurobasal medium, N2 supplement, B27 without vitamin A, 1% GlutaMAX, 1% minimum essential media-nonessential amino acid, 0.1% β-mercaptoethanol, 2.5 μg/ml insulin (Sigma-Aldrich), 200 ng/ml laminin (Sigma-Aldrich), 100 ng/ml SHH-C25II, and 100 ng/ml FGF8. Once the hMO were embedded in Matrigel, a more expanded neuroepithelium began to form in the organoids. To promote growth and differentiation, the hMO were transferred into ultra-low-attachment 6-well-plates (Costar) containing the final organoid differentiation media (Neurobasal medium, N2 supplement, B27 without vitamin A, 1% GlutaMAX, 1% minimum essential media-nonessential amino acid, 0.1% β-mercaptoethanol, 10 ng/ml BDNF [Peprotech], 10 ng/ml GDNF [Peprotech], 100 μM ascorbic acid [Sigma-Aldrich], and 125 μM db-cAMP [Sigma-Aldrich]). The hMO were cultured using an orbital shaker to enhance nutrients and oxygen exchange. Antibiotics (1% of P/S [Life Technologies]) were included in the culture media to prevent potential bacterial contamination over a long-term culture. The medium was replenished every 2 days.

HCO were generated as previously described^47^. HiPSCs were dissociated with accutase to single cells and were plated (10^4^ cells/well) in low-attachment, U-shaped 96-well plates with 200 µl/well of mTeSR supplemented with FGF2 (4 ng/ml; R&D Systems) and ROCK inhibitor (50 µM). Cells were cultured for 48 h to form uniformly sized EBs. Half of the media was aspirated and 150 µl/well of fresh mTeSR supplemented with FGF2 (4 ng/ml) and ROCK inhibitor (50 µM) was added. After 48 h (day 4 of the organoid generation), half of the media was aspirated and 150 µl/well of fresh mTeSR without ROCK inhibitor and FGF2 was added. At day 7, EBs were transferred to a low-attachment 24-well plate and fed with 500 µl/well DMEM/F12 media supplemented with 1% of N2, 1% of minimum essential media-nonessential amino acids, and 1 µg/ml Heparin every second day to form neuroectodermal spheroids. At day 11, each neuroectodermal spheroid was embedded in 15 µl of Matrigel to promote 3D growth and organization, incubated at 37 °C for 20 min to allow solidification of Matrigel, and transferred to ultra-low-attachment 6-well plate with 2 ml of Cortical Differentiation Media (CDM) consisting of 50:50% of Neurobasal:DMEM/F12 media mixure supplemented with 0.5% of N2, 1% of B27 (no vitamin A), 0,5% of minimum essential media-nonessential amino acids, 0,1% of β-mercaptoethanol, 1% P/S, and 0,025% of insulin (Peprotech). Media was half-changed every other day and starting from day 15, B27 without vitamin A was replaced with B27 with vitamin A. Organoids were cultured on an orbital shaker starting from day 11.

### Culture of human astrocytes

Human cerebellar astrocytes were cultured according to the manufacturer’s instructions. Briefly, 5000 astrocytes/cm² were cultured in astrocyte medium with supplements (ScienCell Research Laboratories) in uncoated T75 flasks or 6-well plates at 37 °C, 5% CO_2_. Full media changes were performed every three days, and astrocytes were passaged after reaching approximately 90% confluency using trypsin/EDTA treatment for 3 min at RT. To assess MHC class I expression, astrocytes were treated with 1 µg/ml LPS for 24 h to induce an inflammatory response and fixed with 4% paraformaldehyde (PFA, Invitrogen) for 15 min at 37 °C.

### T cells isolation and activation

T cells were isolated from PBMCs of healthy Caucasian individuals using a Pan T Cell Isolation Kit (Miltenyi Biotec) and magnetic isolation according to the manufacturer’s instructions. T cells were cultured in RPMI medium (Thermo Fischer), GlutaMAX Supplement (Life Technologies) with 10% heat-inactivated Fetal Calf Serum (FCS; Thermo Fischer). T cells were frozen in 90% KOSR (Thermo Fisher) with 10% DMSO (Invitrogen) at a rate of −1 °C/min using a Mr. Frosty freezing container and were transferred into the liquid nitrogen at the next day. To thaw T cells, cryovials containing the cells were placed into a water bath at 37 °C for 1 min. The thawed cells were transferred into pre-warmed DMEM (Life Technologies) and centrifuged at 500 × *g* for 5 min. Cells were resuspended in pre-warmed RPMI/10% FCS and transferred onto a culture plate. T cells were activated ex vivo using CD3/CD28 dynabeads (Thermo Fisher) for 48 h to achieve a polyclonal T cell activation by triggering TCRs and the costimulatory molecule CD28 by antibodies against CD3 and CD28, respectively. After 48 h, beads were detached from the cells by vortexing for 1 min and separated from the cells on the magnet prior to the co-culture of T cells with organoids.

### Development of allogeneic co-culture of hMO and T cells

To determine an optimal co-culture condition, which would be suitable for both hMO and T cells, we cultured T cells and day 30 hMO generated from two control hiPSCs lines in four different co-culture media: hMOM, hMOM + IL-2 (Sigma-Aldrich), SFLM (Life Technologies), and SFLM + IL-2. hMOM represented the best conditions for hMO. SFLM is developed for the optimal serum-free growth and expansion of human T lymphocytes ex vivo, and due to the absence of serum, it is suitable for organoids. Moreover, hMO supplements were added to this medium to support hMO viability. IL-2 promotes T cells survival and its addition to either hMOM or SFLM is supportive for T cells in the co-culture.

Moreover, hMO supplements (1:100 N2 supplement, 1:50 B27 without vitamin A, 1% GlutaMAX, 1% minimum essential media-nonessential amino acid, 0.1% β-mercaptoethanol, 10 ng/ml BDNF, 10 ng/ml GDNF, 100 μM ascorbic acid, and 125 μM db-cAMP) were added to SFLM to support hMO viability. IL-2 was used in a concentration of 25 U/ml. Both hMO and T cells were cultured separately in four different co-culture media for 7 days and then harvested for further analyses. For co-cultures, organoids (day 30 hMO or day 60 hMO) were transferred to 24-well plate containing 2 ml of hMOM with IL-2, and co-cultured with T cells at a ratio of 2 organoids per 1 million of T cells (in 1 well) for 7 days at 37 °C, 5% CO2 on an orbital shaker.

### Organoid tissue dissociation

To analyze infiltrating T cells, hMO were dissociated to a single-cell suspension by using Miltenyi Papain-based neural tissue dissociation kit (Miltenyi Biotech). Organoids were transferred to a 15 ml tube and washed with Hanks’ Balanced Salt Solution (HBSS). Enzyme mix 1 consisting of 50 µL of Papain (enzyme P) and 1900 µL of Buffer X per sample was prepared. 1950 µL of enzyme mix 1 was added to each tube and incubated at 37 °C for 15 min. Enzyme mix 2 consisting of 10 µL of DNase (enzyme A) and 20 µL of Buffer Y per sample was prepared. 30 µL of enzyme mix 2 was added to each tube and tubes were shacked manually. Tissue pieces were triturated 5–10 times with 1000 μl wide-bore and P1000 pipette tips. The tissue pieces were incubated twice for 10 min at 37 °C with trituration steps in between and after with P1000 and P200 pipette tips. Depending on the amount of tissue clumps, additional 5 min of incubation might have been necessary. Cells were filtered through a 40 µm strainer and centrifuged at 300 × *g* for 5 min. Cells were resuspended in HBSS and viability and cell counts were assessed using an Acridine Orange/Propidium Iodide Stain (Logos Biosystem) on the automated fluorescence cell counter LUNA™ (Logos Biosystem).

### T cell reactivation

For reactivation, cells were stimulated ex vivo with 50 ng/mL phorbol 12-myristate 13-acetate (PMA, Sigma-Aldrich) and 1 µg/mL ionomycin (Sigma-Aldrich) in the presence of protein transport inhibitor 1 µg/mL monensin (GolgiStop™, BD Biosciences), to allow intracellular cytokine accumulation. The reactivation was performed in 24-well culture plates in a total volume of 2 mL at 37 °C with 5% CO_2_ for 4 h. After stimulation, cells were washed with PBS, stained for surface and intracellular markers, and analyzed by flow cytometry.

### Flow cytometry analysis

For flow cytometry analysis, live-dead staining was performed using LIVE/DEAD™ Fixable Dead Cell Stain Kit (Thermo Fisher Scientific) according to the manufacturer’s instructions. For surface staining, 10^5^ cells/staining were incubated with saturating amounts of fluorescently-labeled antibodies for 15 min at 4 °C in the dark. For intracellular staining, cells were fixed with 4% PFA for 15 min at 37 °C. 0.5 million cells/staining were permeabilized in perm/wash buffer (Thermo Fisher Scientific) for 30 min on ice, blocked with 10% FCS and Fc receptor block (Miltenyi Biotec) in perm/wash buffer for 60 min on ice, and incubated with saturating amounts of fluorescently labeled antibodies for 60 min on ice in the dark. All antibodies used for flow cytometry analyses are listed in the Table 1. Flow cytometry was performed using Cytoflex flow cytometer and analyzed using CytExpert software (both from Beckman Coulter, Inc.). Fluorescence signals were determined using an appropriate electronic compensation to exclude emission spectra overlap.

**Table 1:** Antibodies used for flow cytometry analyses

Antigen	Isotype	Supplier	Clone	Catalog №
CD3 APC/Cyanine7	mouse IgG1	BioLegend	UCHT1	300425
CD3 Alexa Fluor 488	rat IgG2b k	BioLegend	17A2	100210
CD25 APC	recombinant human IgG1	Miltenyi Biotec	REA570	130-113-284
CD4 PE	mouse IgG2aκ	Miltenyi Biotec	VIT4	130-113-214
CD8 APC	mouse IgG2aκ	Miltenyi Biotec	BW135/80	130-098-078
LFA-1 PerCP/Cyanine5.5	Rat IgG1, κ	BioLegend	H155-78	141008
VLA-4 α chain BV421	Mouse IgG1, κ	BioLegend	9F10	304322
VLA-4 β chain PE/Cyanine5	Mouse IgG1, κ	BioLegend	TS2/16	303006
CXCR4 BV510	Mouse IgG2a, κ	BioLegend	12G5	306536
IL-17 FITC	recombinant human IgG2	Miltenyi Biotec	REA1063	130-118-242
IL-2 PE	recombinant human IgG1	Miltenyi Biotec	REA689	130-111-303
Il-10 APC	recombinant human IgG0	Miltenyi Biotec	REA842	130-112-729
IFN-y PE	recombinant human IgG1	Miltenyi Biotec	REA600	130-113-498
TNF-a BV650	Mouse IgG1, κ	BDBiosciences	MAb11 (RUO)	563418
CD107a Alexa Fluor 488	Mouse IgG1, κ	BioLegend	H4A3	328610
Granzyme B PB	Mouse IgG1, κ	BioLegend	GB11	515408

### Gene expression analysis

For RNA extraction, organoids were washed from Matrigel by using Corning® Cell Recovery Solution (Sigma) and lysed using TRIzol (Invitrogen) at RT for 5 min followed by phenol-chloroform phase separation by centrifugation at 12,000 × *g* for 15 min at 4 °C. Total RNA was isolated from the upper aqueous phase using RNeasy Kit (QIAGEN) according to manufacturer’s instructions with an on-column DNase digestion. 500 ng of total RNA was reversely transcribed into cDNA using the QuantiTect Reverse Transcription Kit (QIAGEN) according to the manufacturer’s instructions and diluted 1:1 with ultrapure water. 1 μl of cDNA was used per qRT-PCR reaction. qRT-PCR was performed as previously described (Sommer et al., 2016). Target genes were analyzed using the following primer pairs (Sigma): FOXG1: forward 5’- AGAAGAACGGCAAGTACGAGA -3’ and reverse 5’- TGTTGAGGGACAGATTGTGGC -3’; EMX: forward 5’- CGCCTTCGAGAAGAACCAC -3’ and reverse 5’- GGTTCTGGAACCACACCTTC -3’; ASCL1: forward 5’- AGGTGGAGACACTGCGCT -3’ and reverse 5’- CGATCACCCTGCTTCCAAAGT -3’; TH: forward 5’- TCCACGCTGTACTGGTTCAC -3’ and reverse 5’- TGTACGGGTCGAACTTCACG -3’; EN1: forward 5’- CGCAGCAGCCTCTCGTATG -3’ and reverse 5’- CCTGGAACTCCGCCTTGAG-3’; SOX2: forward 5’- TACAGCATGTCCTACTCGCAG -3’ and reverse 5’- GAGGAAGAGGTAACCACAGGG -3’; MAP2: forward 5’- CGAAGCGCCAATGGATTCC -3’ and reverse 5’- TGAACTATCCTTGCAGACACCT -3’; TUBB3: forward 5’- -3’ and reverse 5’- -3’; OTX2: forward 5’- CAAAGTGAGACCTGCCAAAAAGA -3’ and reverse 5’- TGGACAAGGGATCTGACAGTG -3’; FOXA2: forward 5’- GGAGCAGCTACTATGCAGAGC -3’ and reverse 5’- CGTGTTCATGCCGTTCATCC -3’; GAPDH: forward 5’- AGCCACATCGCTCAGACAC -3’ and reverse 5’- GCCCAATACGACCAAATCC -3’; B2M: forward 5’- GAGGCTATCCAGCGTACTCC -3’ and reverse 5’- AATGTCGGATGGATGAAACC-3’, RPL0: forward 5’- AGCCCAGAACACTGGTCTC -3’ and reverse 5’- ACTCAGGATTTCAATGGTGCC -3’.

### Immunocytochemistry (ICC)

Organoids were fixed in 4% PFA in DPBS (Invitrogen) for 30 min at RT, incubated in a 30% sucrose (Carl Roth) solution in DPBS at 4 °C overnight, and subsequently embedded in Tissue-Tek OCT compound (Sakura Finetek) for cryosectioning. Frozen organoids were cryosectioned at a thickness of 12 μm using CM3050 S cryostat (Leica). Organoid cryosections were incubated in the antigen retrieval buffer (Thermo Fisher) at 70 °C for 20 min, washed with DPBS, and blocked with 10% donkey serum, 0.3% TritonX-100, and Fc receptor block in DPBS for 1 h at RT. After blocking, the sections were incubated with primary antibodies overnight, washed with DPBS, and incubated with the appropriate fluorescently-labeled secondary antibodies for 1 h at RT, followed by 2 min incubation with 4’,6-diamidino2-phenylindole DAPI (10 μg/ml; Sigma) at RT to stain the nuclei. All primary antibodies used for ICC are listed in the Table 2. Secondary antibodies for ICC were donkey anti-rat Alexa Fluor 488, anti-chicken and anti-mouse Alexa Fluor 647, anti-rabbit Alexa Fluor 488 and 546, and anti-goat Alexa Fluor 488.

All sections were mounted with Aqua-polymount reagent (Polyscience). Images were taken on the Zeiss Observer Z1 fluorescence microscope (Zeiss) using either a mosaic mode or a z-stack function followed by the extended depth of focus application.

**Table 2:** Antibodies used in ICC

Antigen	Isotype	Supplier	Clone	Catalog №
CD3	rat monoclonal	Bio-Rad	CD3-12	VMA00015
MAP2	ms monoclonal IgG1	SIGMA	HM-2	M9942
SOX2	rb monoclonal	Cell Signaling	D6D9	3579
TH	rb polyclonal	Abcam	n/a	ab112
FoxA2	gt polyclonal IgG	R&D Systems	n/a	AF2400
ICAM1	ms monoclonal IgG	R&D Systems	BBIG-11	BBA3
VCAM1	recombinant rb	Thermo Fisher	SA05-04	MA531965
CXCL12	rb polyclonal	Proteintech	n/a	17402-1-AP
MHC I	ms monoclonal IgG2a	Santa Cruz	F-3	sc-55582

### Cell death analysis

To determine the cell death rate in organoid tissues, after antigen retrieval step, Click-iT™ Plus Terminal deoxynucleotidyl transferase dUTP nick end labeling (TUNEL) Assay for In Situ Apoptosis Detection (Thermo Fischer) was applied according to the manufacturer’s instructions. Afterwards, cryosections were blocked and stained as described above.

### Image analysis

Nuclei and specific markers (DAPI, CD3, TUNEL, and SOX2) were counted by using the CellProfiler 4.2.1^48^. The pipeline for human cell count (available on the website https://cellprofiler.org/examples) was adjusted individually for each marker. The total number of CD3+, TUNEL+ or SOX2+ cells per one cryosection was divided by the total number of DAPI-positive cells on the same cryosection. To assess the neuronal presence in the hMO tissue, the relative MAP2 signal was evaluated. Counting single MAP2-positive cells is not feasible due to the fact that 3D hMO tissue was cryosectioned for ICC. As a result, within one cryosection, we observe somas and neurites from different layers, making it impossible to trace individual neurons. Therefore, we assessed the relative mean fluorescence intensity (MFI) of MAP2 per cryosection normalized to the MFI of the DAPI signal in each cryosection. MFI was measured using the ImageJ 1.8.0 software^49^.

### Statistical analysis

In our study, covariates such as T cell presence in hMO tissue (+T cells vs. no T cells), organoid type (hMO vs. hCO), organoid age (day 30 vs. day 60), and T cell activation status (activated vs. non-activated T cells) were evaluated.

For each experimental condition, n is reported/depicted as single point on a graph as the number of independent organoid cultures or biological replicates used in the analysis. In flow cytometry experiments, n refers to the number of independent T cell samples from different donors or co-cultures. Measurements were taken from distinct samples in all experiments. Each data point represents an independent biological replicate, ensuring no repeated measurements from the same sample within a single dataset.

Assumptions of normality were checked for all datasets using Shapiro–Wilk tests. For datasets not meeting the assumption of normality, non-parametric statistical tests (e.g., Mann–Whitney test or Spearman’s correlation) were used. For central tendency, data are presented as mean ± standard deviation (SD) unless otherwise specified. For datasets with non-normal distributions, the mean was used because of consistency with other analyses or the need for comparability. Differences between two groups were analyzed by an unpaired t test with Welch’s Correction due to unequal variances or unequal sample sizes or by Mann–Whitney test. For more than two groups, data were analyzed by two-way ANOVA. For correlation analyses, Spearman’s rank correlation test (two-sided) was applied to examine relationships between T cell presence and cell death (TUNEL+ cells) or neuronal loss (MAP2 signal). *p* ≤ 0.05 was considered significant. Statistical analyses were performed using GraphPad Prism 8 (GraphPad Software).

## Supplementary information

Supplemental Information Novel Co-culture Model of T Cells and Midbrain Organoids for Investigating Neurodegeneration in Parkinson's Disease

## Data Availability

Data are available from the corresponding author upon reasonable request.
