# Supplementary material for: Novel co-culture model of T cells and midbrain organoids for investigating neurodegeneration in Parkinson’s disease
Source: NPJ Parkinsons Dis. 2025 Feb 28;11:36. doi: 10.1038/s41531-025-00882-8 (PMC11871142; doi:10.1038/s41531-025-00882-8)
Supplement: Supplementary file 1 — Supplemental Information Novel Co-culture Model of T Cells and Midbrain Organoids for Investigating Neurodegeneration in Parkinson's Disease [file 41531_2025_882_MOESM1_ESM.pdf]

Supplemental Information

Novel Co-culture Model of T Cells and Midbrain Organoids for Investigating Neurodegeneration in Parkinson's Disease

Elizaveta Gerasimova, Amke Beenen, Daniil Kachkin, Martin Regensburger, Sebastian Zundler, David B. Blumenthal, Gloria Lutzny-Geier, Beate Winner, and Iryna Prots

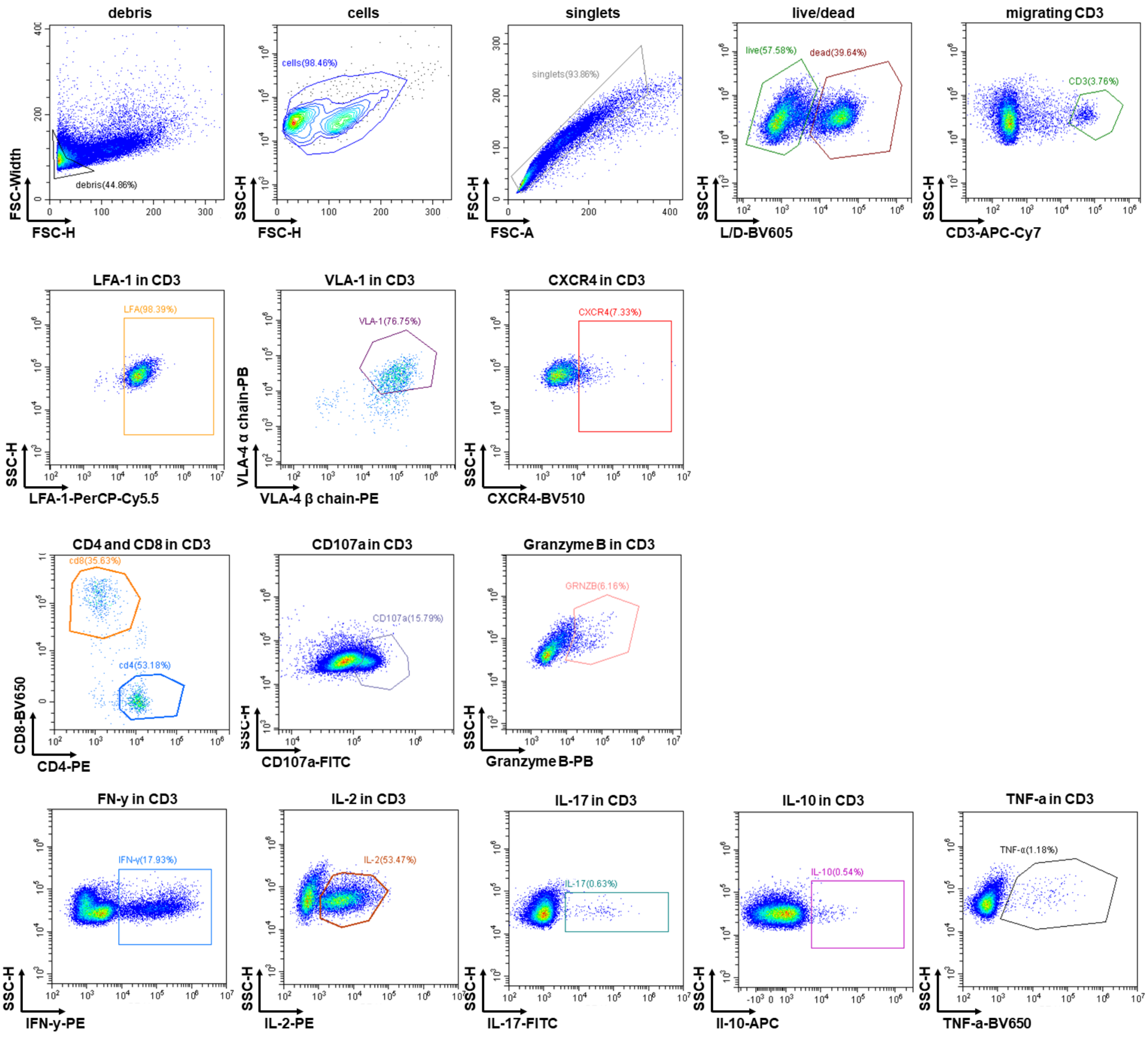

Figure S1 (related to Fig. 4 and 5). **Gating strategy for flow cytometry analysis.** Forward scatter height (FSC-H) versus forward scatter width (FSC-Width) was used to identify and exclude debris, ensuring that only cellular events were included in the analysis. Single cells were identified by plotting forward scatter area (FSC-A) against FSC-H, which allowed for the exclusion of doublets. Live/Dead discrimination was performed using the LIVE/DEAD™ Fixable Dead Cell

Stain Kit, where dead cells were identified by high fluorescence intensity in the viability dye channel and excluded from further analysis. Marker-positive populations, such as CD4+ and CD8+ T cells, were identified based on their respective fluorescently labeled markers.

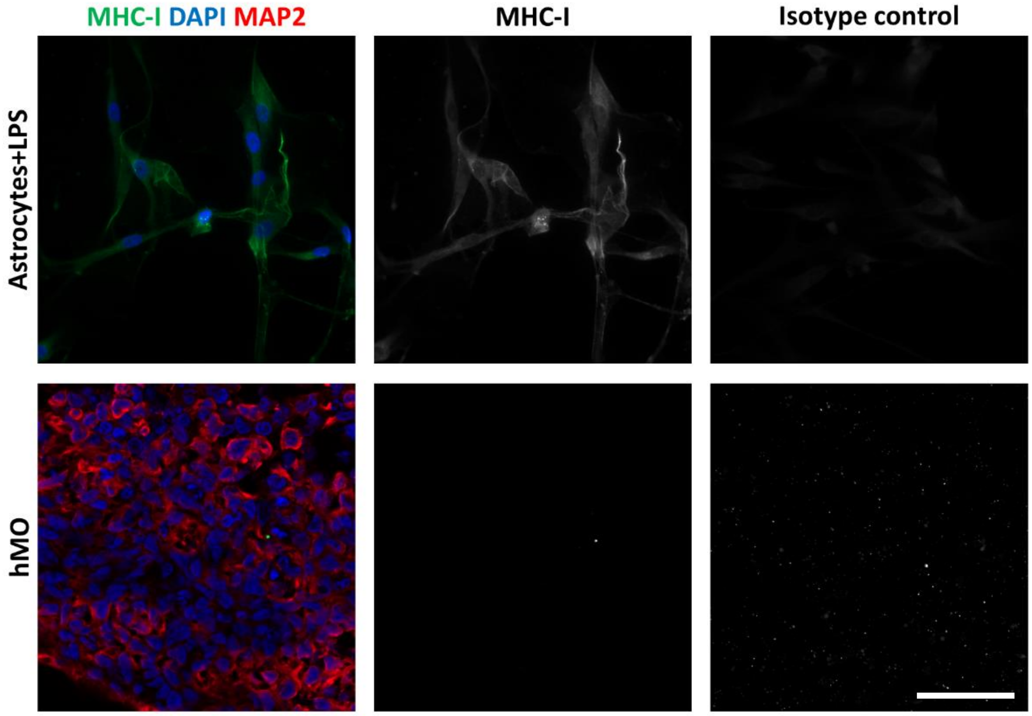

Figure S2. MHC class I in hMO. Immunocytochemistry staining for MHC class I (MHC-I) in hMO has not detected any MHC-I expression. Representative immunostaining of MHC-I (green) and MAP2 (red) in hMO (lower panel) and LPS-treated human astrocytes (Astrocytes+LPS; upper panel). Scale bar = 100 μm.

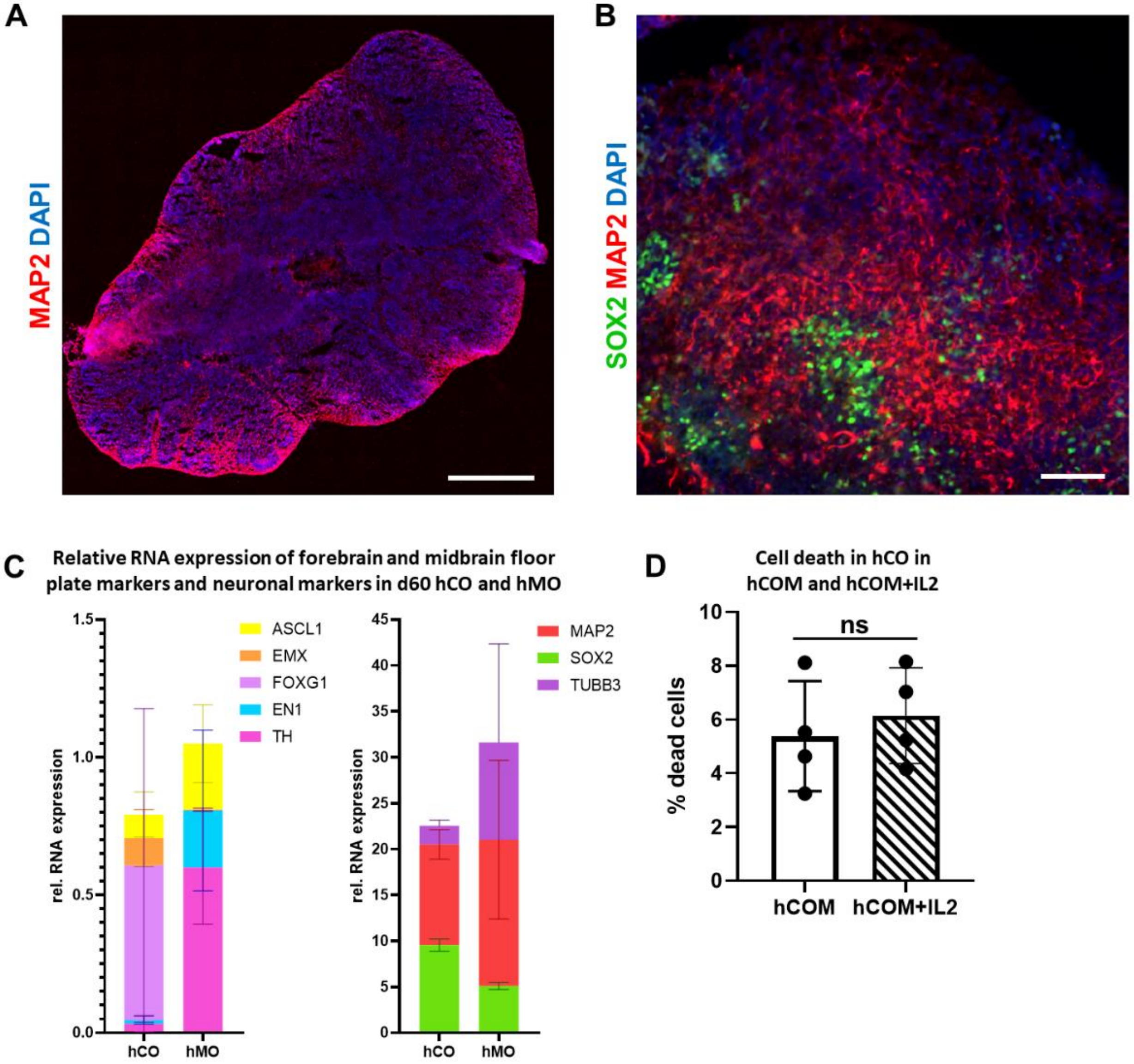

Figure S3 (related to Fig. 6). **Human Cerebral Organoids (hCO) express neuronal markers and have forebrain identity. (A-B)** hCO express neuronal and neuroprogenitor markers. Representative immunostaining of **(A)** MAP2 (red) and **(B)** SOX2 (green) and MAP2 in hMO cryosections at day 60. Scale bars 500µm and 100µm respectively. **(C)** hCO express neuronal markers and forebrain floor plate markers. Left: Relative RNA expression of ASCL1 (yellow), EMX (orange), FOXG1 (light violet), EN1 (light blue) and TH (purple) in hCO in comparison to hMO at day 60. Right: Relative transcript expression of SOX2 (green), MAP2 (red), and β3-tubulin (TUBB3, violet) in hCO in comparison to hMO at day 60. Transcript expression of every marker is normalized to housekeeping genes (HKGs: RPL0, GAPDH, B2M). Bar graphs are stacked to improve clarity. **(D)** Addition of IL-2 to the human Cerebral Organoid Medium (hCOM) did not not increase hCO cell death.
